# Psychological Distress and Presenteeism During the Pandemic Period: A Quantitative Study Among Nurses From Emergency and Critical Care

**DOI:** 10.1155/jonm/4278999

**Published:** 2026-04-17

**Authors:** Xiuwen Mu, Maria Baldonedo-Mosteiro, Mireia Blanco González, Ricardo Baldonedo-Cernuda, Sara Franco-Correia, Maria-Pilar Mosteiro-Díaz

**Affiliations:** ^1^ Wenzhou Medical University Eye Hospital, Wenzhou, Zhejiang, China; ^2^ Medicine Department, Faculty of Medicine and Health Sciences, University of Oviedo, Asturias, Spain, uniovi.es

**Keywords:** critical care nursing, emergency departments, presenteeism, psychological distress

## Abstract

**Aim:**

To assess the psychological status and the prevalence of presenteeism among Spanish nurses working in emergency departments (EDs) and intensive care units (ICUs) and to explore the association between these variables.

**Background:**

Nurses working in ED and ICU settings experience higher levels of psychological stress, including burnout and lower job satisfaction. Anxiety and depression are common in this group and may contribute to nursing presenteeism. Presenteeism is defined as continuing to work despite feeling ill or unwell. There are gaps in research addressing the psychological status and presenteeism of ED and ICU nurses in Spain.

**Methods:**

A cross‐sectional study using the Kessler Psychological Distress Scale (K‐10) and the Stanford Presenteeism Scale (SPS‐6) was conducted between April and December 2022. In total, 182 participants were included. The study followed the STROBE guidelines.

**Findings:**

Psychological distress was identified in 35.7% of participants, and a presenteeism prevalence of 43.8% was observed among nurses. A statistically significant association between psychological distress and presenteeism was found (*p* < 0.001). Age, educational background, and perceived work‐related stress (*p* = 0.05) were identified as factors associated with psychological distress. Perceived work‐related stress was also significantly associated with presenteeism (*p* < 0.05).

**Conclusions:**

More than one‐third of nurses working in ED and ICU settings experienced psychological distress, and a high prevalence of presenteeism was identified. Presenteeism was associated with higher levels of psychological distress.

**Implications for Nursing:**

Early detection of psychological distress and the implementation of workplace interventions may help reduce presenteeism among nurses.

## 1. Introduction

It is well known that under ordinary circumstances, healthcare professionals exhibit high levels of psychological distress as well as presenteeism. When considering COVID‐19, it is easy to understand how this situation may be exacerbated because professionals face additional risks and threats to their work and occupational well‐being [1]. These risks include not only direct exposure to the virus but also conflicts arising from the need to balance professional and family obligations [2]. In any healthcare system, healthcare workers are considered a pillar of sustainability, and the COVID‐19 pandemic made this fact even more evident because their continuous presence was clearly demonstrated. Despite their essential role, healthcare workers have faced the challenge of presenteeism for far too long. Presenteeism is a phenomenon that is particularly prevalent in this group and is defined as the act of working while sick, determined by both cultural expectations and organizational demands [3]. Because of the uncertainty of the circumstances, as well as the high level of risk the situation represented, professionals experienced elevated levels of anxiety, distress, and even job dissatisfaction [4, 5].

## 2. Background

Evidence suggests that in recent years, emergency department (ED) and intensive care unit (ICU) wards worldwide have been highly stressful work environments for nurses [6–8]. Nurses working in these settings experience heavy workloads, burnout, long‐term fatigue, threats of infection, and frustration related to the death of patients under their care [9, 10].

### 2.1. Psychological Distress Among Nurses

Psychological disturbances have been associated with prolonged work‐related stress that affects nurses’ physical and mental health status [7, 11–13], as well as their professional performance. This relationship is often mediated through emotional exhaustion, vicarious trauma, fatigue, and professional burnout [13–15]. Previous data indicate that job stress is a contributing factor to psychological distress and that stress levels among nurses range from moderate to severe, with prevalence estimates between 42% and 77% [16].

One study reported that 90.6% of 337 Spanish nurses working in ED and ICU settings experienced anxiety and depression [14]. Compared to nurses from other departments, those working in ED and ICU environments experience nearly two to seven times higher levels of psychological stress, as shown in a study conducted among Indian nurses [16]. Extended working hours and an endless nursing workload are often unavoidable and may intensify feelings of sadness, depression, and helplessness. As a result, nurses tend to experience psychological distress in an invisible or unrecognized manner.

### 2.2. Presenteeism

Psychological factors in nursing, such as work demands, affect nursing productivity [17] and contribute to the prevalence of presenteeism [18]. Presenteeism is defined as attending work despite being ill [19–21] and represents a risk to public health. Several studies have shown that negative psychological factors are associated with poor mental health and a higher prevalence of presenteeism among nurses [18, 22–24].

Presenteeism among nurses may also lead to poor professional behavior, reduced quality of nursing care, and an increased risk of clinical errors. Female nurses represent the majority of the nursing workforce, and it has been reported that younger nurses may have weaker psychological defenses for coping with job‐related difficulties, placing them at higher risk of mental exhaustion [25].

At the same time, the nursing workforce worldwide is facing population aging and a persistent labor shortage [26, 27]. Compared to other professions, nursing requires specialized knowledge, theoretical training, and technical skills, as well as the ability to respond appropriately to emergency situations. Clearly, not everyone is able to replace nurses in clinical roles. In addition, the pandemic context and occupational characteristics have resulted in nurses continuing to work on the frontline even when they are ill or require time for recovery. Recent research conducted in Spain found that it is both sensible and valuable to reduce presenteeism behaviors (e.g., depression, anxiety, and insomnia) by promoting psychological resilience, such as increased job satisfaction [28]. However, this study lacked detailed descriptions of nurses’ current psychological status.

### 2.3. Purpose

The purpose of this study is to establish the relationship between nurses’ mental and psychological status and the prevalence of presenteeism, a topic that is novel in this professional group, given the timing of data collection. This study of nurses’ psychological distress and presenteeism during the pandemic contributes to a critical understanding of the “vicious cycle” linking mental health to productivity loss at work. Its primary contribution is to highlight that psychological distress is likely a significant predictor of presenteeism, which in turn leads to measurable declines in patient safety and quality of care. The investigators believe that understanding the relationship between these two factors may provide valuable information not only for ICU nursing but also for general nursing wards internationally. In addition, the investigators aim to address specific research questions: Is psychological distress a predictor of presenteeism? Are there individual characteristics or profile features that may help identify professionals who are more likely to experience presenteeism and psychological distress during a crisis such as a pandemic?

### 2.4. Study Aims

This study assesses the psychological status and prevalence of presenteeism among Spanish ED and ICU nursing staff and explores the association between these variables.

## 3. Methods

### 3.1. Research Design

A cross‐sectional, quantitative study was conducted in accordance with the STROBE guidelines. The research period extended from April to December 2022.

### 3.2. Sample and Setting

Data were collected in the ED and ICU of Hospital Universitario Central de Asturias (HUCA), a general hospital located in Oviedo, the administrative capital of Asturias (Spain). HUCA is a tertiary‐level hospital dedicated to research, teaching, patient care, and the provision of community health services for the public.

All nursing staff working in the ED and ICU at the selected setting were considered the study population. Based on a population of 303 nurses and assistant nurses and using a sample size calculator with a confidence level of 95%, a margin of error of 5%, and a population proportion of 50%, a required sample size of 170 participants was estimated. The inclusion and exclusion criteria were defined by the researchers based on previous studies. The inclusion criteria were being a nursing assistant or a registered nurse working in the ED or ICU for at least 1 month and agreeing to participate through informed consent. The nursing management model in Spain is relatively unique, involving frequent rotation of nurses across different contexts and mobility between services within a hospital, between hospitals, or even across levels of care (hospital and community healthcare). As a result, nurses may work only a limited number of shifts in a given unit. This organizational context justified the methodological decision to include nurses with a minimum of 1 month of experience in the unit. Incomplete questionnaires were excluded from the analysis. In total, 187 questionnaires were distributed, and 182 valid responses were obtained, constituting a convenience sample. All returned surveys were anonymized.

### 3.3. Variables and Instruments

#### 3.3.1. Sociodemographic and Labor Variables

Sociodemographic variables included sex, age, marital status, presence of children, and educational level. Labor‐related variables were also collected, including work unit, type of contract, shift type, total nursing experience, and experience working in the ED and critical care area. In addition, participants were asked about their perception of work‐related stress and whether they engaged in other activities after their shift.

#### 3.3.2. Psychological Distress Scale (K‐10)

The 10‐item Kessler Psychological Distress Scale (K‐10) was used to assess psychological distress. This brief instrument measures nonspecific psychological distress and has been widely adopted by clinicians to screen for common mental disorders, including anxiety and depression [29]. The Spanish‐language version of the scale was used in this study [30]. A Cronbach’s alpha of 0.901 was reported in the Spanish validation study. Participants were asked to reflect on psychological changes experienced during the previous 30 days. Responses are scored on a 5‐point Likert‐type scale ranging from 1 (*none of the time*) to 5 (*all of the time*), yielding a total score between 10 and 50. Lower scores indicate lower levels of psychological distress, while higher scores indicate greater distress. Scores of 10–19 represent low psychological distress, 20–24 mild distress, 25–29 moderate distress, and 30–50 severe psychological distress (Australian Bureau of Statistics [31, 32]). In this paper, we considered scores ≥ 20 as significant psychological distress, which mean that all levels are considered except the low level.

#### 3.3.3. Stanford Presenteeism Scale (SPS‐6)

The validated Spanish version of the SPS‐6 [33] was used to assess the prevalence of presenteeism. The adaptation and validation study conducted in Spain included 495 healthcare workers and reported Cronbach’s alpha values of 0.873 for Factor 1 and 0.826 for Factor 2 [33]. The original version of the instrument was developed by Koopman et al. [34]. The prevalence of presenteeism was assessed using the question: “During the last month, have you shown up for work despite feeling sick?” [19]. Participants who answered “yes” were classified as exhibiting presenteeism and were asked to complete the remaining six items of the scale. Those who answered “no” were classified as not exhibiting presenteeism. All six items are rated on a 5‐point Likert scale.

Because the SPS‐6 and K‐10 assess experiences over a similar time frame, this study is the first to combine the Spanish versions of both instruments to explore psychological distress levels in relation to the prevalence of presenteeism.

### 3.4. Data Collection

After receiving approval from the Ethics Committee and permission from the hospital Board, the aim of the study was explained to the staff, and printed questionnaires were distributed face‐to‐face to participants in the ED and ICU units. Regarding the data collection procedure, the investigators chose to use printed instruments delivered directly to eligible participants. The main reason for this methodological choice was that during the pandemic period, healthcare professionals were being asked to complete a large number of surveys and questionnaires, which could negatively affect response rates.

During each rotating shift, nurses and assistant nurses were invited to participate. Questionnaires were distributed and, approximately 30 min later, the researcher returned to collect the completed forms. If the researcher was unable to collect a questionnaire during a participant’s shift, participants were asked to place the completed questionnaire in a closed envelope at the end of their shift.

### 3.5. Ethical Considerations

Ethical approval was obtained from the Research Ethics Committee of the Principality of Asturias (Spain) (No. 102/18). Before distributing the instruments, the researcher informed participants from the three clinical areas about the study objectives. To protect participants’ identities and ensure confidentiality, all responses were anonymized. Each participant who agreed to take part in the study signed an informed consent form after receiving information from the researcher. The consent form was submitted together with the completed questionnaire and included information regarding data use and sharing. No incentives or financial compensation were offered for participation.

### 3.6. Data Analyses

All data were entered and analyzed using the Statistical Package for the Social Sciences (SPSS) (Version 25.0). The Kolmogorov–Smirnov test was used to assess the normality of the data distribution. Descriptive statistics, including frequencies, means, and standard deviations (SDs), were used to describe participant characteristics. Although the sample size calculation was based on the total nursing staff in selected settings, and the study recruited nurses and nursing assistants as participants, the investigators decided not to perform an independent analysis considering two different samples. We therefore considered nursing staff in the research results and discussion.

Cross‐tabulation using the chi‐square test was applied to analyze associations between scale variables and nominal variables. The Kruskal–Wallis test was used to examine relationships between levels of psychological distress and independent variables. The Mann–Whitney U test assessed associations between presenteeism and independent variables, as well as the correlation between the prevalence of presenteeism and levels of psychological distress. Finally, a multivariate logistic regression model was then constructed, including all variables identified by the researchers as potential predictors. A stepwise selection algorithm was applied, and the final model is presented in the last column of the table (multivariate odds ratio).

All *p* values of < 0.05 were considered statistically significant.

## 4. Results

### 4.1. Participant Characteristics

The sample consisted of 182 participants, with a response rate of 86%.

The majority of participants were female (91.2%). Of the total sample, 94 participants (52.2%) were married. The mean age was 39.5 years (SD = 9.7). The participants had a mean of 13.6 years (SD = 9.0) of professional nursing experience and 6.8 years (SD = 7.4) of experience working in the ED or ICU. Eighty‐eight participants (48.4%) had a permanent contract, and 157 (86.3%) worked rotating shifts. Overall, 152 nurses (83.5%) reported feeling stressed at work, and 151 (83.9%) reported engaging in activities after work. Table 1 presents the characteristics of the study sample.

**TABLE 1:** Sample characteristics.

Variable	Category		Frequency		Percent	
Unit	ED		**75**		41.2	
	ICU		107		58.8	

Sex	Female		**166**		91.2	
	Male		16		8.8	

Marital status	Single		74		41.1	
	Married/partner		**94**		52.2	
	Divorced		10		5.6	
	Widowed		2		1.1	

Child	Yes		77		42.3	
	No		**105**		57.7	

Educational background	Vocational training		44		24.2	
	Diplomate/graduate		**80**		44.0	
	Master		37		20.3	
	Specialist		21		11.5	

Contract type	Part‐time job		38		20.9	
	Temporary		56		30.8	
	Permanent		**88**		48.4	

Shift type	Morning		6		3.3	
	Morning/afternoon		11		6.0	
	Morning/afternoon/night		**157**		86.3	
	Slide		8		4.4	

Feeling work to be stressful	Yes		**152**		83.5	
	No		30		16.5	

Activities after work	Yes		**151**		83.9	
	No		29		16.1	

**Variable**	***n***	**Min**	**Max**	**Mean**	**SD**	**Missing**

Age, years	177	24	63	39.5	9.7	5
Years of professional experience	181	0.1	38.0	13.6	9.0	1
Years in ED and critical care areas	176	0.1	38.0	6.8	7.4	6

*Note:* The bold value indicates the most frequent category.

Abbreviations: ED, emergency department; ICU, intensive care unit; SD, standard deviation.

### 4.2. Psychological Distress Levels and Presenteeism Prevalence

Significant psychological distress was identified in 60 participants (35.7%), with 30 (20.2%) presenting mild levels of distress and 19 (11.3%) showing moderate levels. Regarding presenteeism among nurses working in the ED and ICU, a prevalence of 43.8% was observed (Table 2).

**TABLE 2:** Psychological distress levels and presenteeism prevalence.

	***n***	**%**

Psychological distress level		
Low (10–19)	108	64.3
Mild (20–24)	34	20.2
Moderate (25–29)	19	11.3
Severe (≥ 30)	7	4.2
Presenteeism		
Yes	78	43.8
No	100	56.2

### 4.3. Correlation Analyses

#### 4.3.1. K10 Scores vs. Sociodemographic and Labor Variables

Correlation analyses showed that education level and perceived work‐related stress were statistically associated with psychological distress (*p* = 0.030 and *p* = 0.012, respectively). Nurses with a vocational training educational level reported higher psychological distress scores than nurses with other educational levels. In addition, participants who perceived their work as stressful exhibited higher levels of psychological distress than those who did not report this perception (Table 3).

**TABLE 3:** Association between psychological distress and sociodemographic and labor factors.

Variable	Category	Psychological distress level					Total	*p*
		Missed	Low	Mild	Moderate	Severe		
Unit	ED	4	43	16	10	2	75	0.918
	ICU	10	65	18	9	5	107	

Sex	Female	12	98	32	17	7	166	0.798
	Male	2	10	2	2	0	16	

Marital status	Single	7	50	8	5	4	74	0.106
	Married/partner	6	49	23	13	3	94	
	Divorced	0	8	1	1	0	10	
	Widowed	1	0	1	0	0	2	

Child	Yes	5	43	18	9	2	77	0.585
	No	9	65	16	10	5	105	

Education level	Vocational training	6	16	12	8	2	44	0.030^∗^
	Diplomate/graduate	7	53	10	8	2	80	
	Master	1	24	6	3	3	37	
	Specialist	0	15	6	0	0	21	

Contract type	Part‐time job	5	21	5	4	3	38	0.636
	Temporary	4	34	12	4	2	56	
	Permanent	5	53	17	11	2	88	

Shift type	Morning	2	3	0	1	0	6	0.470
	Morning/afternoon	0	8	2	1	0	11	
	Morning/afternoon/night	11	93	29	17	7	157	
	Slide	1	4	3	0	0	8	

Feel work to be stressful	Yes	12	82	33	19	6	152	0.012^∗^
	No	2	26	1	0	1	30	

Engage in other activities after working	Yes	13	94	23	16	5	151	0.099
	No	1	13	10	3	2	29	

Abbreviations: ED, emergency department; ICU, intensive care unit.

^∗^ *p* < 0.05.

**FIGURE 1:**
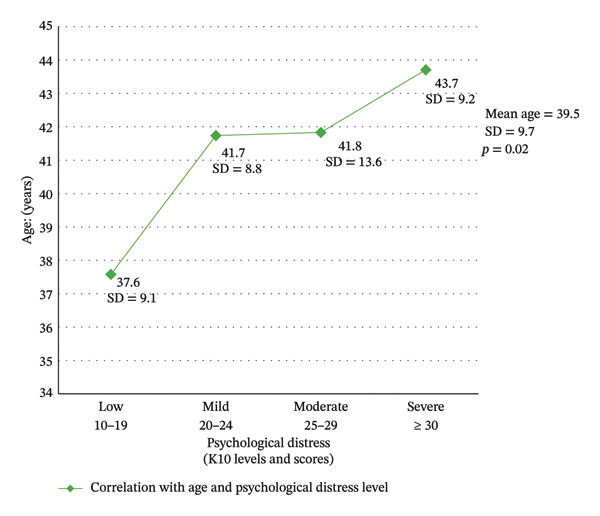
Relationship between psychological distress levels and age.

A statistically significant association was also found between psychological distress and age (*p* = 0.02). Nurses younger than 38 years showed low levels of psychological distress. As nurses’ mean age increased, the level of psychological distress also increased. Mild and moderate levels of psychological distress were concentrated among nurses between 41 and 42 years of age (Figure 1).

#### 4.3.2. Presenteeism vs. Sociodemographic and Labor Variables

The same correlational analysis was conducted to examine the relationship between presenteeism and sociodemographic and labor variables. Among these variables, only perceived work‐related stress was significantly associated with presenteeism (*p* = 0.006). Most participants who exhibited presenteeism also reported feeling that their work was stressful. No significant associations were found between presenteeism and the remaining sociodemographic or labor variables. Table 4 represents the multivariate logistic regression model.

**TABLE 4:** Presenteeism regression model.

		**Presenteeism**		**Univariate OR**	**Multivariate OR**
		**No presenteeism**	**Presenteeism**		

Unit	ER	36 (53.7)	31 (46.3)	—	—
	ICU	52 (57.9)	38 (42.2)	0.85 (0.45–1.61, *p* = 0.613)	—

Sex	Female	82 (56.9)	62 (43.1)	—	—
	Male	6 (46.2)	7 (53.8)	1.54 (0.49–5.01, *p* = 0.456)	—

Marital status	Divorced	5 (55.6)	4 (44.4)	—	—
	Married/partner	41 (49.4)	42 (50.6)	1.53 (0.81–2.91, *p* = 0.194)	—
	Single	42 (64.6)	23 (35.4)	0.68 (0.17–3.00, *p* = 0.598)	—

Children	No	55 (60.4)	36 (39.6)	—	—
	Yes	33 (50.0)	33 (50.0)	1.53 (0.81–2.91, *p* = 0.194)	—

Education	Diplomate/graduate	39 (56.5)	30 (43.5)	—	—
	Master	19 (54.3)	16 (45.7)	1.09 (0.48–2.48, *p* = 0.828)	
	Specialist	14 (70.0)	6 (30.0)	0.56 (0.18–3.20, *p* = 0.447)	—
	Vocational training	16 (48.5)	17 (51.5)	—	—

Contract type	Part‐time	22 (64.7)	12 (35.3)	—	—
	Permanent	40 (54.1)	34 (45.9)	1.56 (0.68–3.58, *p* = 0.292)	3.14 (1.02–10.16, *p* = 0.049)
	Temporary	26 (53.1)	23 (46.9)	1.62 (0.67–4.06, *p* = 0.292)	2.03 (0.78–5.54, *p* = 0.154)

Shift type	Morning	2 (50.0)	2 (50.0)	—	—
	Morning/afternoon	5 (55.6)	4 (44.4)	0.80 (0.07–9.33, *p* = 0.853)	—
	Morning/afternoon/night	81 (56.2)	63 (43.8)	0.78 (0.09–6.63, *p* = 0.804)	—

Feeling work to be stressed	No	23 (82.1)	5 (17.9)	—	—
	Yes	65 (50.4)	64 (49.6)	4.53 (1.74–14.14, *p* = 0.004)	5.22 (1.95–16.79, *p* = 0.002)

Practice of any activity after work	No	15 (55.6)	12 (44.4)	—	—
	Yes	73 (56.2)	57 (43.8)	0.98 (0.42–2.28, *p* = 0.955)	—

Age, years	Mean (SD)	39.2 (9.6)	39.9 (10.0)	1.01 (0.97–1.04, *p* = 0.666)	1.04 (1.00–1.09, *p* = 0.002)

Total professional experience	Mean (SD)	13.6 (9.5)	13.4 (8.3)	1.00 (0.96–1.03, *p* = 0.850)	—

Experience in the current unit	Mean (SD)	7.0 (8.1)	6.3 (6.3)	0.99 (0.94–1.03, *p* = 0.538)	—

The simplified model explained 12% of the variability in presenteeism and yielded a significant likelihood ratio test (*χ* ^2^(4) = 14.76). No evidence of collinearity was observed, as assessed by variance inflation factors (all < 5), and the model demonstrated an area under the curve of 0.663. The final model included age (because of its proximity to the significance threshold), contract type (nurses with permanent contracts were more likely to exhibit presenteeism than those with part‐time contracts; OR = 3.14, *p* = 0.049), and perceived work‐related stress (OR = 5.22, *p* = 0.002).

#### 4.3.3. Psychological Distress Levels vs. Presenteeism

A statistically significant difference was identified between psychological distress and presenteeism (*p* < 0.005). Higher levels of psychological distress were observed among nurses who reported presenteeism (Table 5).

**TABLE 5:** Relationship between prevalence of presenteeism and mean K‐10 score.

		**Mean K-10 score**	***n***	**SD**	**p**

Presenteeism	Yes	2.1	78	0.6	**< 0.001** ^∗^
	No	1.7	100	0.5	
	Total	1.9	178	0.6	

*Note:* Bold value indicates statistical significance.

^∗^Student’s *t*‐test.

## 5. Discussion

Nursing represents the largest workforce across most health systems. The literature indicates that nurses belong to a group of healthcare professionals who have been particularly affected by psychological distress. Given that these professionals worked under high‐stress settings and demanding conditions during the COVID‐19 pandemic [35–37], it is important to devote further attention to exploring this reality within the Spanish context. Globally, available data suggest that nurses working in ED and ICU settings experience notable psychological distress. In the present study, 35.7% of the sample reported some level of psychological distress. Lower prevalence rates were reported in a study of 455 Canadian ICU healthcare workers, in which the mean total K‐10 score was compatible with psychological distress among nurses, physicians, and other healthcare professionals; however, nurses reported any degree of psychological distress (mild, moderate, or severe) more frequently (23%) than other professional groups [38]. These K‐10 findings are lower than those observed in the present study. Higher levels of psychological distress have been reported elsewhere [39], and a substantially higher prevalence was described in a study of 264 nurses, in which 9.1% presented severe psychological distress [40]. Nevertheless, it is important to acknowledge that nurses were exposed to extraordinary working conditions during the pandemic, which may have exacerbated or worsened preexisting psychological distress. This deterioration not only poses risks to nurses’ mental and psychological health but may also compromise patient safety and the quality of nursing care.

Female sex has often been suggested as a factor associated with work‐related psychological distress among nurses. In the present study, 83.5% of nurses reported feeling that their work was stressful, and nearly the entire sample was female; however, no statistically significant association was observed. Tuckett et al. [41] similarly reported that female nurses were more likely to experience work‐related exhaustion, although no significant differences were found between psychological distress and sex. By contrast, a study involving 445 medical students reported significantly higher K‐10 scores among female students compared to males (*p* = 0.011) [42].

The present study showed that perceived work‐related stress was significantly associated with psychological distress. Nurses with higher K‐10 scores were also more likely to report feeling that their work was stressful. This finding is consistent with a previous study of 623 nurses, in which perceived stress was positively associated with psychological distress [43]. These results reinforce the idea that high occupational or work‐related stress has a direct and measurable impact on psychological distress levels.

It is also important to note that stress and mental health among healthcare workers became particularly prominent topics during the COVID‐19 pandemic. During the first wave of the outbreak, studies showed that healthcare staff experienced high levels of stress in addition to anxiety and depression [44, 45]. Similarly, a study of 821 Portuguese nurses examining the association between mental health promotion strategies and symptoms of depression, anxiety, and stress during the COVID‐19 outbreak found high levels of stress among participants. Interestingly, lower stress levels were observed among nurses working in mental health settings [46].

In the present study, a statistically significant association was also observed between psychological distress and educational background (*p* < 0.03). Nurses with vocational training reported higher levels of psychological distress than those with other educational backgrounds. On one hand, this finding aligns with results reported by Azizoğlu et al. [25]. On the other hand, research conducted in the general population has shown that individuals with low or medium educational levels may have a lower risk of psychological distress than those with higher educational attainment [47].

Aging influences nurses’ general health and may lead to social dysfunction and reduced work engagement. Our analyses indicated that the mean age of nurses working in ED and ICU settings was statistically associated with psychological distress (*p* = 0.02). Specifically, older nurses reported higher levels of psychological distress. By contrast, a recent study of 264 nurses that also used the K‐10 scale found a significant negative relationship between age and work experience and psychological distress, with older nurses reporting lower levels of distress [40].

Attending work despite being ill is a characteristic particularly associated with nursing and has important implications at multiple levels [48]. In the present study, a substantial prevalence of presenteeism was observed (43.8%), although higher prevalence rates have been reported elsewhere. A study conducted among nurses in the same Spanish region found that more than 50% of ED nurses exhibited presenteeism [49]. In a multicenter study including 659 nurses from Portugal, Spain, and Brazil, Portuguese nurses reported the highest prevalence of presenteeism, followed by Brazilian and Spanish nurses. The authors also noted that nurses with less professional experience showed lower levels of presenteeism [50]. Similarly, a study of 388 Chinese nurses reported that 74% exhibited presenteeism, resulting in a 32.86% loss in nursing productivity [51]. In the context of the COVID‐19 pandemic, it is particularly important to establish additional associations to better understand the true scope of the problem. An Australian study found that despite exhibiting COVID‐19 symptoms, 20% of healthcare workers continued to attend work [52]. Given the specific transmission characteristics of COVID‐19, such behavior had a direct impact on regional infection rates.

Data analyses in the present study showed that presenteeism was associated with age, with a higher prevalence observed among older nurses. Previous research has similarly indicated that employees aged 40 years and older are more likely to exhibit presenteeism than younger workers [53]. These findings suggest that mental health interventions should be implemented as early as possible to address this issue.

Psychological distress and presenteeism also appear to be closely correlated. This study is among the few that have simultaneously examined psychological distress and presenteeism in nursing staff. The results showed that higher levels of psychological distress were present among nurses who reported presenteeism (*p* < 0.01). A Canadian study identified associated factors that could be incorporated into a predictive model of presenteeism and psychological distress, in which psychological distress was strongly associated with presenteeism and explained 35% of the variance in the model [54].

Presenteeism may be influenced by a wide range of personal and organizational factors. Qualitative research using focus groups with Portuguese and Swiss frontline nurses during the COVID‐19 pandemic explored factors associated with presenteeism and reported that some nurses “*referred to how they had attended work when unwell and had received positive feedback for their conscientiousness, their commitment to the job and the organization, and for having spared their team members from having to replace them*” [55], p. 12.

Assessing current psychological distress among clinical nurses is essential. On the one hand, it allows nurses and managers to gain a clearer understanding of nurses’ mental health status. On the other hand, such results may serve as a reference point for determining whether psychological interventions are needed. One previous study emphasized that the SPS‐6 primarily detects the consequences of presenteeism but does not help identify the underlying factors contributing to presenteeism among nurses [56]. As a result, conditions such as depression or anxiety may be overlooked and further exacerbated. However, recent research suggests that the SPS‐6 may also be useful in detecting psychological distress. Lower scores on the second dimension of the SPS‐6 (Items 2, 5, and 6) have been associated with better psychological status [57] and lower levels of burnout [33].

This study also clarified that while independent factors change score by score, presenteeism has a greater impact on psychological distress than psychological distress has on presenteeism. This finding suggests that a lower occurrence of presenteeism contributes to better psychological health. These results are consistent with previous research on nursing presenteeism and occupational health [18, 22, 23, 58].

Results of the nonparametric tests showed that only age (*p* = 0.009) and working experience in the ED and ICU (*p* = 0.037) were correlated with psychological distress. According to these findings, additional psychological interventions may be needed for nurses with longer working experience in ED and ICU settings to reduce presenteeism.

Regardless of the variety of psychological factors associated with presenteeism, psychological assessment measures should be implemented to reduce presenteeism among nurses. To promote nurses’ mental health, the theory of planned behavior may provide useful guidance for nursing managers [59]. Managers can offer social support to help reduce job‐related stress and presenteeism [60], and future research should follow nurses who have undergone psychological interventions to evaluate their effects on presenteeism.

Although this study was conducted during the pandemic, it focused on frontline healthcare staff. It would be valuable to replicate this study in the same professional group using a larger sample of nurses from different countries with similar cultural characteristics in order to better understand the true impact of the pandemic on presenteeism and psychological distress.

### 5.1. Limitations

Although this study produced relevant findings, several limitations should be acknowledged. First, data collection coincided with summer and winter vacation periods. Because of seasonal patterns, many nurses tend to take leave during these times, which meant that not all ED and ICU nurses at the study site were able to participate. In addition, the study was conducted in a single Spanish hospital, limiting the generalizability of the findings to other settings. Further longitudinal research involving multiple hospitals in Spain is recommended to examine presenteeism prevalence while incorporating psychological interventions.

The authors are also aware of the theoretical limitations associated with this study. Although some research has examined nursing psychological distress and presenteeism separately, little is known about the relationship between these phenomena during the pandemic, particularly among Spanish ICU nurses. A major limitation is the lack of prior research, as this topic remains relatively new and existing literature is limited. As a result, the research team was required to develop new conceptual approaches rather than building on well‐established theoretical frameworks. From a design perspective, it should also be noted that the cross‐sectional nature of the study does not allow for causal inferences, unlike longitudinal designs.

### 5.2. Implications for Nursing Management and Future Research

The findings of this study highlight the need for systematic assessment of psychological distress among nursing staff, as it appears to be associated with presenteeism. Addressing nurses’ psychological well‐being may therefore have a direct impact on productivity and the quality of patient care.

By identifying relevant psychological factors, the results suggest that implementing organizational structures that promote motivation and professional accomplishment may help reduce psychological distress and, consequently, presenteeism. Potential interventions by managers and leaders include promoting healthy behaviors, encouraging healthy lifestyles, and supporting work–family balance. These strategies are likely to have positive effects on organizational engagement, professional satisfaction, and the overall work environment. Future research should evaluate the impact of such interventions on both presenteeism prevalence and psychological distress among nurses.

Greater attention to nurses’ psychological status is essential as part of efforts to reduce presenteeism. Policies aimed at preventing psychological distress in nursing are needed. Given the limitations associated with conducting this study in a single hospital, multicenter studies, including settings outside Spain, are recommended to explore different healthcare contexts and evaluate human resource management models that may improve presenteeism rates and nurses’ psychological and emotional well‐being.

## 6. Conclusions

This study identified notable levels of psychological distress and a substantial prevalence of presenteeism among nurses. Nurses with vocational training reported higher levels of psychological distress, although few participants experienced severe distress. Less than half of ED and ICU nurses reported presenteeism. Higher levels of psychological distress were observed among nurses who exhibited presenteeism, with a statistically significant association.

An association was found between psychological distress and educational level, with nurses holding vocational training qualifications experiencing higher distress than those with other educational backgrounds. Participants who perceived their work as stressful reported higher levels of both psychological distress and presenteeism. Age was also significantly associated with psychological distress, with nurses over 40 years of age reporting higher levels. These findings suggest that increased psychological support may be particularly important for healthcare workers in their mid‐40s.

Overall, this study contributes to a better understanding of the nursing profiles most likely to experience psychological distress and presenteeism during or following a health crisis. The findings also clarify the relationship between psychological distress and presenteeism among nurses working in high‐risk settings that were heavily exposed during the COVID‐19 pandemic.

## Funding

This study received no specific grant from any funding agency in the public, commercial, or not‐for‐profit sectors.

## Ethics Statement

This study was approved by the Research Ethics Committee of the Principality of Asturias (CeimPA 102/15).

## Conflicts of Interest

The authors declare no conflicts of interest.

## Data Availability

The data that support the findings of this study are available from the corresponding author upon reasonable request.
